# Iodoform Bituminate

**Published:** 1889-03

**Authors:** 


IODOFORM BITUMINATE.—This new iodoform preparation recently introduced by Dr. S. Ehrmann, instructor at the University of Vienna and assistant surgeon in the Vienna clinic for syphilis, is now being manufactured on a commercial scale and our first supplies will soon be in stock. Dr. Ehrmann, in a recent issue on the "Centralblatt fur die gesammte Therapie," publishes a lengthy dissertation on the successful application of Iodoformium bituminatum, from which we take the following description:

"Iodoformium bituminatum resembles mica, in transparent scales of a brownish, metallic, glistening appearance, can be easily powdered, and is totally void of characteristic iodoform odor, retaining only a faint, not unpleasant, smell of tar. It has been used principally in the treatment of ulcers of the skin, by the application of the powder with a brush."

